# UPLC-MS/MS-Based Rat Serum Metabolomics Reveals the Detoxification Mechanism of Psoraleae Fructus during Salt Processing

**DOI:** 10.1155/2021/5597233

**Published:** 2021-09-14

**Authors:** Dan Wang, Na Li, Shengrong Li, Yilong Chen, Leilei He, Xiaomei Zhang

**Affiliations:** Chongqing Engineering Research Center of Pharmaceutical Evaluation of Chinese Medicine, Chongqing Academy of Chinese Materia Medica, Chongqing 400065, China

## Abstract

Psoraleae Fructus (PF) is a botanical medicine widely used in Asian countries, of which salt products have higher safety and efficacy. However, the biological mechanism of the detoxification of salt-processing Psoraleae Fructus (SPF) has not yet been revealed. In this study, UPLC-MS/MS technology was used to explore the metabolic differences between SPF and PF in normal rats and reveal the mechanism of salt processing. The histopathological results of rat liver and kidney showed that the degree of liver and kidney injure in the SPF group was less than that in the PF group. The results of metabolomics showed that the detoxification mechanism of PF by salt processing might be related to glycerophospholipid metabolism, phenylalanine, tyrosine, and tryptophan biosynthesis, arginine and proline metabolism, phenylalanine metabolism, and linoleic acid metabolism. PF-induced inflammation could be reduced by regulating the expression of metabolites to achieve the purpose of salt processing and detoxification. It included reducing the production of metabolites such as 1-acyl-sn-glycero-3-phosphocholine, sn-glycero-3-phosphocholine, tyrosine, arginine, linoleic acid, arachidonic acid, and phenylacetylglycine/hippuric acid ratio and upregulating the expression of metabolites such as creatine.

## 1. Introduction

Psoraleae Fructus (PF) is the dried and mature fruit of the legume *Psoralea corylifolia* L., which has the functions of warming kidney and assisting yang, promoting qi absorption to calm panting, and warming the spleen and check diarrhea. Modern studies have found that PF has anti-inflammatory [1], antibacterial [2], antioxidant [3], antitumor [4], and similar estrogen-like effects [5]. It has complex therapeutic potential and is worthy of our in-depth study. At present, the commonly used Chinese medicine decoction pieces are PF and salt-processing Psoraleae Fructus (SPF), both of which are used in the treatment of osteoporosis [6], fractures, and vitiligo [7] through compatibility with other Chinese medicines. Clinical applications have found that, compared with PF, SPF not only has better curative effects on diseases such as osteoporosis but also shows less liver and kidney toxicity relatively [8, 9].

Drug processing is a major feature of Chinese medicine. Common processing methods include steaming in water, salt processing, sand blanching, vinegar frying, and rice wine stewing [10]. According to the theory of traditional Chinese medicine, these processing methods can improve the efficacy of drugs, reduce toxicity, change the channel tropism of the original herbal medicine, or improve the smell and taste of the drugs [11]. At present, related research on the effect of SPF is mainly focused on the changes of active chemical components in vitro [12]. Research on the changes of metabolomics in vivo after salt processing is still lacking. And the metabolism of drugs in vivo is the key to revealing changes in the efficacy or toxicity of drugs.

Metabolomics provides an overview of the overall metabolism in organisms through high-resolution analysis and stoichiometric statistical tools [13, 14]. It can not only develop reliable biomarkers through identification but also accurately predict and evaluate the safety of drugs [15, 16]. It can also analyze the pharmacological and toxicological characteristics of the drug in depth, explain the metabolic pathways that may be involved in regulation in the organism, and provide unique insights for drug toxicological research [17]. Therefore, based on metabolomics technology, normal rats were used as the research carrier to explore the metabolic differences before and after PF processing, to systematically and comprehensively reveal the internal mechanism of salt processing to reduce the liver toxicity of PF. At the same time, it further proved the scientific connotation of traditional Chinese medicine processing.

## 2. Materials and Methods

### 2.1. Reagents and Animals

Standard of L-2-chlorophenylalanine was purchased from Shanghai Hengbai Biotechnology Co., Ltd. (Shanghai, China). Reagents of methanol, acetonitrile, ammonium acetate, and formic acid were purchased from CNW technologies (Shanghai, China). PF was identified as the dry mature fruit of legume *Psoralea corylifolia* L. by researcher Li Longyun from Chongqing Academy of Chinese Medicine. The samples werekept in Chongqing Research Institute of Traditional Chinese Medicine.

18 SPF male Sprague-Dawley rats, 180 ∼ 200 g, were provided by Laboratory Animal Research Institute of Chongqing Institute of Traditional Chinese Medicine, certificate number SCXK (Chongqing) 2012-0006. The rats were reared in the room with a temperature of 23 ± 1°C and relative humidity of 40%–60%. The circadian rhythm is normal. They were free to drink and eat. The animal experiments involved in this study were approved by the Animal Experiment Ethics Committee of Chongqing Institute of Traditional Chinese Medicine.

### 2.2. Extraction

PF extract was prepared by adding 8 times of petroleum ether to PF and refluxing extraction twice, 1 hour each time, filtering and combining the filtrate, recovery of petroleum ether by rotary evaporation, and concentrating the extract to the concentration of 5 g/ml (based on the original medicine).

SPF extract was prepared by adding 2.10 g of salt water solution for every 100 g of PF, mixing well and moistening for 12 h, stir-frying at 80°C for 30 min, and taking it out and cooling to room temperature to obtain SPF. SPF extraction method was the same as PF.

### 2.3. Animal Grouping and Administration

Eighteen healthy male SD rats were adaptively fed for 7 days and randomly divided into the control group, PF group, and SPF group with 6 rats in each group. The rats in the PF group received PF extract 30 g/kg/d, and the rats in the SPF group received SPF extract 45 g/kg/d. The control group was given the same amount of saline vehicle. The duration of administration was 14 days. Rats were free to eat and drink during administration.

### 2.4. Sample Collection

After 12 h of the last treatment, the rats were sacrificed and blood samples were collected from the heart. The blood samples were centrifuged at 3000 rpm for 10 min after standing at room temperature for 1 h. The supernatant was taken out and centrifuged at 12000 speed for 10 min at 4°C. After centrifugation, the serum was taken out and stored in the tube at −80°C. Next, the liver and kidney tissues were excised and fixed in 10% neutral-buffered formalin for 24 h.

### 2.5. Histological Examination

All fixed liver and kidney tissues were embedded in paraffin. Subsequently, the paraffin blocks were cut into sections (about 4∼5 *μ*m thick) and stained with hematoxylin-eosin (HE) as described previously [18].

### 2.6. Serum Sample Processing

Pipette 100 *μ*L of serum sample into EP tube, add 300 *μ*L of methanol (including internal standard 1 *μ*g/mL), and vortex for 30 s to mix. The samples were placed in ice water for 10 min and then at −20°C for 1 h. All samples were centrifuged at 4°C for 15 min at 12000 rpm. Take the supernatant and filter it, put it into the sample bottle, and test it on the machine. All the samples were mixed with the same amount of supernatant to prepare quality control (QC) samples for testing.

### 2.7. Sample Testing

LC-MS/MS analyses were performed using a UHPLC system (1290, Agilent Technologies) with a UPLC HSS T3 column (2.1 mm × 100 mm, 1.8 *μ*m) coupled to *Q* Exactive mass spectrometer (Orbitrap MS, Thermo). The mobile phase A was 0.1% formic acid in water for positive mode and 5 mmol/L ammonium acetate in water for negative mode, and the mobile phase B was acetonitrile. The elution gradient was set as follows: 0∼1.0 min, 1% B; 1.0∼8.0 min, 1%∼99% B; 8.0∼10.0 min, 99% B; 10.0∼10.1 min, 99%∼1% B; 10.1∼12 min, 1% B. The flow rate was 0.5 mL/min. The injected volume was 2 *μ*L. The QE mass spectrometer was used for its ability to acquire MS/MS spectra on information-dependent acquisition (IDA) mode in the control of the acquisition software (Xcalibur 4.0.27, Thermo). In this mode, the acquisition software continuously evaluates the full scan MS spectrum. The ESI source conditions were set as follows: sheath gas flow rate as 45 Arb, aux gas flow rate as 15 Arb, capillary temperature 400°C, full MS resolution as 70000, MS/MS resolution as 17500, collision energy as 20/40/60 eV in NCE mode, and spray voltage as 4.0 kV (positive) or −3.6 kV (negative), respectively.

### 2.8. Data Processing and Analysis

The raw data were converted to the mzXML format using ProteoWizard and processed with an in-house program, which was developed using *R* and based on XCMS, for peak detection, extraction, alignment, and integration. Then an in-house MS2 database (BiotreeDB) was applied in metabolite annotation. The cutoff for annotation was set at 0.3.

The total ion current (TIC) of each sample was used for normalization. After obtaining the sorted data, SIMCA software (V15.0.2, Sartorius Stedim Data Analytics AB, Umea, Sweden) was used to transform the data into logarithmic (log) and centralization (CTR) format and then perform principal component analysis and modeling.

The first principal component is modeled and analyzed by OPLS-DA. The quality of the model was tested by 7-fold cross validation. Then the validity of the model was evaluated by *R*^2^*Y* (the interpretability of the model to the classified variable *Y*) and *Q*^2^ (the predictability of the model). Finally, through the permutation test, the order of the categorical variable *Y* is randomly changed multiple times to obtain different random *Q*^2^ values, which further test the validity of the model.

In the OPLS-DA model, metabolites with variable importance in the projection (VIP) greater than 1 and *P*-value of Student's *t*-test less than 0.05 were selected as differential metabolites. Volcano plot was used for visualization. The differential metabolites were clustered by the complete linkage method and displayed in a heatmap. The differential metabolites were submitted to the Kyoto Encyclopedia of Genes and Genomes (KEGG) pathway database (https://www.kegg.jp/kegg/pathway.html). The possible metabolic pathways were analyzed.

### 2.9. Statistical Analysis

IBM SPSS Statistics 20 software was used for statistical analysis. The measurement data were expressed as mean ± standard deviation (SD). The data were analyzed using multivariate statistical analysis methods and univariate analysis (UVA) methods such as analysis of variance (ANOVA) and Student's *t*-test. *P* < 0.05 indicates that the difference was statistically significant, and *P* < 0.01 indicates that the difference was extremely significant.

## 3. Results

### 3.1. Histological Examination

**Figure 1:**
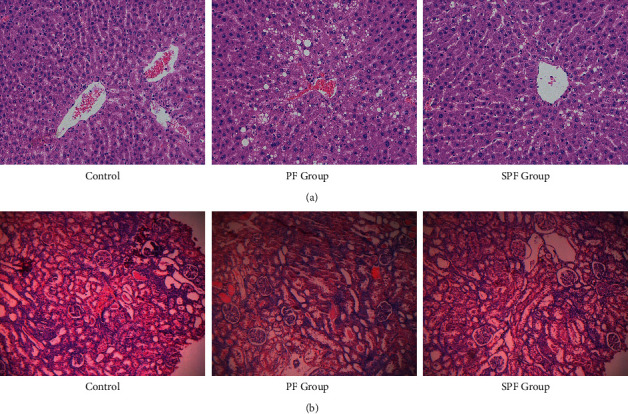
Effects of PF and SPF on the morphology of liver and kidney in rats (HE staining × 100). (a) Liver tissue. (b) Kidney tissue.

HE staining was used to observe the changes in liver and kidney of rats in PF and SPF groups. As shown in Figure 1(a), the liver structure of rats in the control group was clear, the cells were arranged orderly, and no obvious pathological changes were observed. The liver tissue of the PF group showed pathological changes such as hepatocyte edema and vacuoles, accompanied by the proliferation of bile duct epithelial cells. Compared with the PF group, the edema, degeneration, and vacuoles of hepatocytes in the SPF group had significantly reduced, and there was no obvious bile duct epithelial cell proliferation. In Figure 1(b), no obvious pathological changes were found in the renal histopathology of the control group. In the PF group, pathological changes such as renal tubular epithelial cell degeneration, cytoplasmic vacuolation, and nuclear pyknosis were seen in the kidney. Protein tubular lesions appeared in the renal tubules. Compared with the PF group, the renal protein tubular lesions in the SPF group were significantly reduced, but there were still tubular epithelial cell lesions in the renal tubules. Pathological results confirmed that PF and SPF had toxicity to liver and kidney. Compared with the liver and kidney damage caused by PF, the liver and kidney toxicity of SPF was reduced.

### 3.2. Quality Control

#### 3.2.1. Process Quality Control

As shown in Supplementary Figures 1(a) and 1(b), the retention time and signal intensity of the QC sample BPC chromatographic peak overlap very well, indicated that the instrument is very stable. As shown in Supplementary Figures 1(c) and 1(d), the retention time and response intensity of the internal standard L-2-chlorophenylalanine in the sample were stable. It showed that the stability of data acquisition was very good.

#### 3.2.2. Data Quality Control

**Figure 2:**
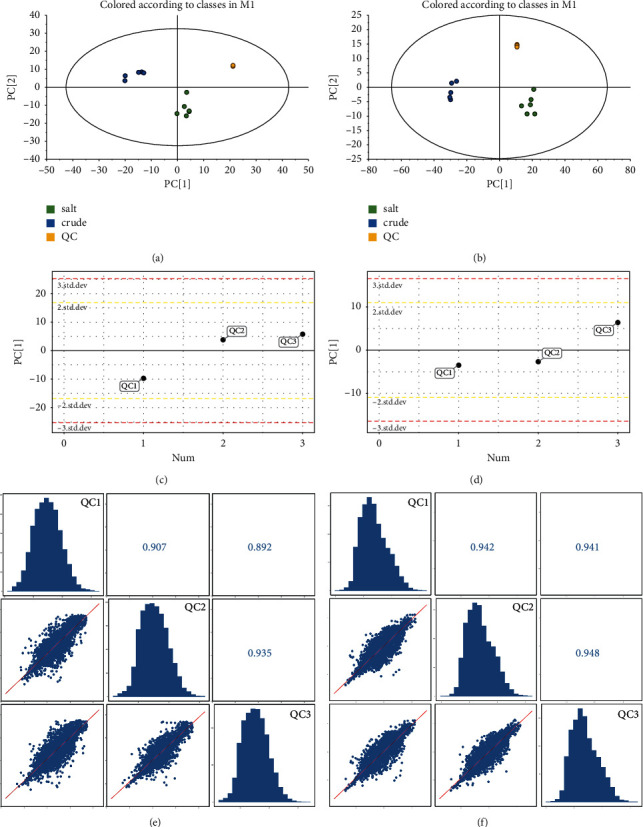
Score scatter plot for PCA model TOTAL with QC. (a) In positive mode. (b) In negative mode. PCA-X one-dimensional distribution of QC sample. (c) In positive mode. (d) In negative mode. Correlation analysis of QC samples. (e) In positive mode. (f) In negative mode.

In Figures 2(a)–2(d), all QC samples were within ± 2 STD. In Figures 2(e) and 2(f), and the correlations of all QC samples were greater than 0.7. All these indicated that the method was stable and the data was reliable.

The internal standard was a substance introduced by an external source. All QC samples had the same internal standard concentration. Therefore, the smaller the response difference of the internal standard (RSD ≤ 20%), the more stable the system and the higher the data quality. The data in Table 1 confirmed that the data quality of this experiment was very high.

### 3.3. Principal Component Analysis (PCA)

**Figure 3:**
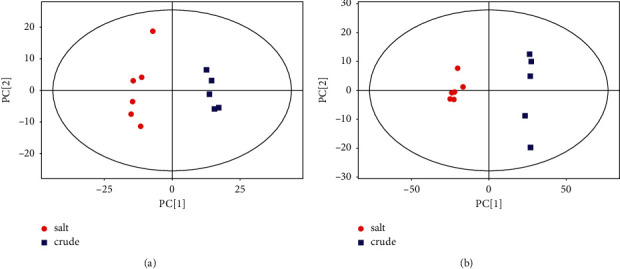
Score scatter plot of PCA model for group salt versus crude. (a) In positive mode. (b) In negative mode.

In Figure 3, the abscissa PC [1] and the ordinate PC [2] represented the scores of the first and second principal components, respectively. The red circle represented the SPF group, and the blue square represented the PF group. The principal component analysis (PCA) results showed that, in the positive and negative modes, the samples were all within the 95% confidence interval (Hotelling's T-squared ellipse), and the clusters between the SPF group and the PF group had significant classifications.

### 3.4. Orthogonal Partial Least Squares Method-Discriminant Analysis (OPLS-DA)

**Figure 4:**
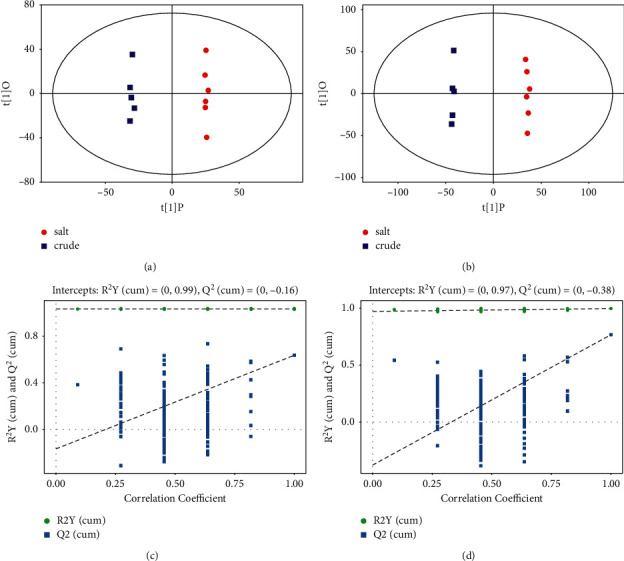
Score scatter plot of OPLS-DA model for group salt versus crude. (a) In positive mode. (b) In negative mode. Permutation test of OPLS-DA model for group salt versus crude. (c) In positive mode. (d) In negative mode.

Since the PCA model could not be used for better visualization and subsequent analysis, we used Orthogonal Partial Least Squares Method-Discriminant Analysis (OPLS-DA) to obtain the metabolic differences between the two groups. The scatter plot results of OPLS-DA (Figures 4(a) and 4(b)) showed that all samples were within the 95% confidence interval (Hotelling's T-squared ellipse), and the SPF group and the PF group were distinguished significantly.

The permutation test (*n* = 200) was used to test the model overfitting and evaluate the statistical significance of the model. In Figures 4(c) and 4(d), the abscissa represented the replacement retention of the replacement test, and the ordinate represented the value of *R*^2^Y or *Q*^2^. The green dot represented the *R*^2^*Y*, the blue square dot represented the *Q*^2^, and the two dashed lines represented the regression lines of *R*^2^*Y* and *Q*^2^, respectively. In the positive and negative modes, *R*^2^*Y* was 0.99 and 0.97, and *Q*^2^ was −0.16 and −0.38, respectively. Those indicated that the mode had good robustness and there was no overfitting phenomenon.

### 3.5. Univariate Statistical Analysis

**Figure 5:**
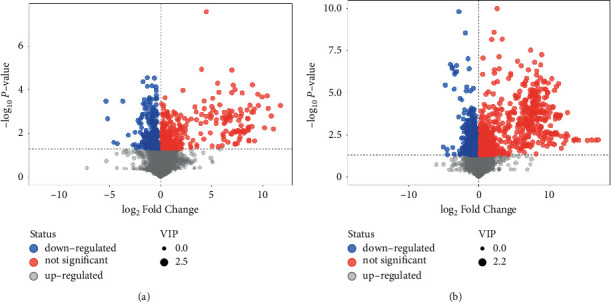
Volcano plot for group salt versus crude. (a) In positive mode. (b) In negative mode.

Univariate statistical analysis was used to screen for metabolic differences. Under the positive and negative modes, 669 and 1495 candidate values were screened out based on the principles of VIP > 1 and *P* < 0.05, respectively. Volcano graph was used to visualize the results (Figure 5). Each point in the volcano graph represented a metabolite. The abscissa represented the multiple change of each substance in the group, and the ordinate represented the *P*-value of Student's *t*-test. The scatter size represented the VIP value of the OPLS-DA model. The larger the scatter, the greater the VIP value. The color of the scatter spot represented the changing trend of metabolites. Red represented significant upregulation of metabolites, blue represented significant downregulation, and gray represented no significant difference.

The candidate metabolites were qualitatively analyzed by secondary mass spectrometry. 43 potential metabolic differences were obtained in positive mode and 24 in negative mode (Supplementary Tables 1 and 2). Subsequently, the potential metabolic differences were matched in the HDMB database and the KEGG database, and the compounds that could be accurately matched were selected as the metabolic differences (Supplementary Tables 3 and 4). In positive and negative modes, 12 metabolic differences were obtained, respectively. As listed in Table 2, the 12 metabolic differences in positive mode were creatinine (C00791), creatine (C00300), trigonelline (C01004), dibutyl phthalate (C14214), tyrosine (C00082), palmitoylethanolamide (C16512), L-palmitoylcarnitine (C02990), 1-acyl-sn-glycero-3-phosphocholine (C04230), aminocaproic acid (C02378), xanthine (C00385), glycerophosphocholine (C00670), and arginine (C00062). As listed in Table 3, the 12 metabolic differences in the negative mode were tryptophan (C00078), phenylacetylglycine (C05598), norleucine (C01933), *β*-guanidinopropionic acid (C03065), linoleic acid (C01595), xanthine (C00385), arachidonic acid (C00219), tyrosine (C00082), oleic acid (C00712), hippuric acid (C01586), creatinine (C00791), and 3,7-dimethyluric acid (C16360).

### 3.6. Metabolites Comparison

**Figure 6:**
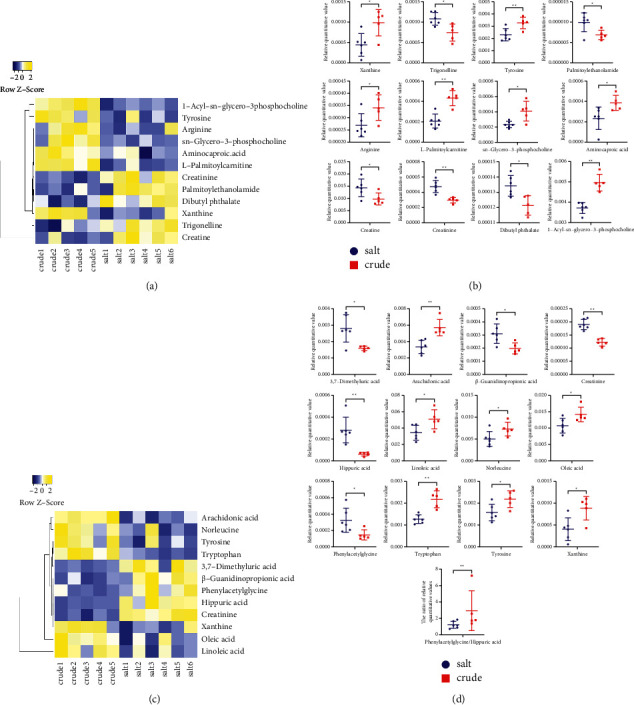
The heatmap and the relative content trends of potential metabolites in the salt group and crude group. (a, b) In positive mode. (c, d) In negative mode.

The relative expression levels of the metabolic differences obtained in the two modes were compared. The Euclidean distance matrix was calculated according to the quantitative values of the differential metabolites between the two groups, and the differential metabolites were clustered by the complete linkage method. As shown in Figures 6(a) and 6(c), the abscissa represents different experimental groups. The ordinate represents the different metabolites. The color patches in different positions represent the relative expression of metabolites. The results showed that, compared with the PF group, 5 metabolic differences were upregulated and 7 metabolic differences were downregulated in the SPF group in positive mode. Five metabolic differences were upregulated and 7 metabolic differences were downregulated in the SPF group in negative mode. Student's *t*-test analysis showed that, compared with the PF group, the relative expression of the differential metabolites creatinine, creatine, trigonelline, dibutyl phthalate, palmitoylethanolamide, phenylacetylglycine, *β*-guanidinopropionic acid, hippuric acid, and 3,7-dimethyluric acid in the SPF group was significantly upregulated. And the relative expression of the differential metabolites tyrosine, L-palmitoylcarnitine, 1-acyl-sn-glycero-3-phosphocholine, aminocaproic acid, xanthine, sn-glycero-3-phosphocholine, arginine, tryptophan, norleucine, hippurate, arachidonic acid, tyrosine, and oleic acid was significantly downregulated (Figures 6(b) and 6(d)).

### 3.7. KEGG Pathway Analysis

**Figure 7:**
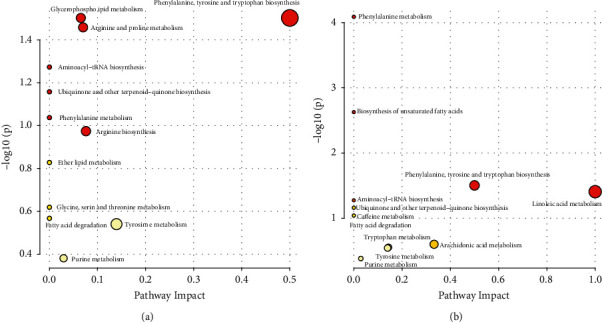
Pathway analysis for group salt versus crude. (a) In positive mode. (b) In negative mode.

KEGG pathway annotation analysis was performed on the differential metabolites. The results show that there were 12 pathways related to metabolite differences in the positive mode and 11 pathways in the negative mode. As shown in Figure 7, each bubble in the bubble chart represented a metabolic pathway. The abscissa of the bubble and the bubble size indicated the size of the path influencing factors in the topology analysis. The larger the scale, the larger the impact factor. The ordinate of the bubble and the bubble color indicated the *P*-value of the enrichment analysis (take the negative natural logarithm, i.e., −ln (*p*)). The darker the color, the smaller the *P* value and the more significant the degree of enrichment. The relevant information was listed in Tables 4 and 5. The key metabolic pathways included glycerophospholipid metabolism, phenylalanine, tyrosine and tryptophan biosynthesis, arginine and proline metabolism, phenylalanine metabolism, and biosynthesis of unsaturated fatty acids.

## 4. Discussion

PF is a widely used Chinese medicine, containing bioactive components such as coumarin, benzofuran, flavonoids, and monoterpenes [12]. Due to clinical application, it has been found that PF has a toxic effect on the liver [9], and animal studies have shown that PF has hepatotoxicity and nephrotoxicity to normal rats [19, 20]. Traditional Chinese medicine processing technology is an effective means to reduce the toxicity of traditional Chinese medicine. SPF, as a processed product of PF, has a higher frequency of clinical application. In the past ten years, the research on the mechanism of reducing toxicity after pf processing has increased. However, studies mostly focus on chemical composition changes [21, 22], and there is a lack of research on its metabolic differences in vivo. Therefore, we explored the potential mechanism of salt processing to reduce PF toxicity from the perspective of metabolism in vivo through animal serum metabolomics technology. Firstly, we compared the effects of PF and SPF on the liver and kidney of rats through pathological observation. The results showed that the liver and kidney damage caused by SPF was reduced compared with the PF group. It is confirmed that salt processing can reduce the toxic injury induced by PF in rats.

Subsequently, we further explored the possible mechanism of salt processing to reduce the toxicity of PF by screening metabolic differences and analyzing metabolic pathway. The results showed that the detoxification of PF by salt processing may be related to the regulation of the following metabolic pathways, including glycerophospholipid metabolism, phenylalanine, tyrosine, and tryptophan biosynthesis, arginine and proline metabolism, phenylalanine metabolism, and linoleic acid metabolism. Different metabolites mainly involved in the above metabolic pathways include 1-acyl-sn-glycero-3-phosphocholine (C04230), sn-glycero-3-phosphocholine (C00670), tyrosine (C00082), arginine (C00062), creatine (C00300), hippuric acid (C01586), phenylacetylglycine (C05598), linoleic acid (C01595), and arachidonic acid (C00219).

### 4.1. Glycerophospholipid Metabolism

Pathway analysis showed that glycerophospholipid metabolism was highly correlated with PF metabolism. Lipids not only constitute most of the cell membrane bilayer but also regulate a variety of biological processes, such as cell proliferation, apoptosis, immunity, angiogenesis, and inflammation [23–25]. Abnormal lipid metabolism is related to the pathogenesis of many human diseases, such as diabetes, obesity, cancer, and Alzheimer's disease [26, 27]. 1-Acyl-sn-glycero-3-phosphocholine (C04230) and sn-glycero-3-phosphocholine (C00670), as differential metabolites involved in glycerophospholipid metabolism, were significantly reduced in the SPF group. 1-Acyl-sn-glycero-3-phosphocholine, namely, lysophosphatidylcholine (LPC), was a well-known inflammatory lipid that is elevated in a variety of inflammation-related diseases [28, 29]. LPC enhances or even induces cell proliferation, stimulates lymphocyte adhesion and differentiation, activates T lymphocytes, and promotes macrophage mitosis through different signal pathways (such as NF-*κ*B, PKC, and ERK) [30]. LPC can induce the expression of cyclooxygenase type 2 (COX-2) through p38/CREB or ATF-1 pathways in vascular endothelial cells, which was a key proinflammatory mediator [31, 32]. COX-2 could catalyze arachidonic acid to various classes of bioactive proinflammatory lipids, such as thromboxanes and prostaglandins [33], which provides additional clues about the role of lysoglycerophospholipids in inflammatory responses. Therefore, the mechanism of detoxification by PF processing may be related to inhibiting the expression of LPC in the glycerophospholipid metabolism pathway and reducing the body's inflammatory response.

### 4.2. Arginine and Proline Metabolism

Arginine and proline metabolic pathway is one of the key metabolic pathways of PF, which is important for the regulation of immune function, urea synthesis, and NO synthesis. In the metabolic pathway of arginine and proline, the relative content of the central metabolite arginine and end products (creatine, creatinine) changed significantly. Arginine plays a key role in various metabolic processes under normal and disease states, including urea cycle, poly amino acid and creatine synthesis, immune function regulation, and NO synthesis [34]. Creatine and creatinine participate in metabolic pathways such as energy metabolism and urea cycle. The liver is the main place for the biosynthesis of creatine in the body. It is finally exported from the liver and transported through the blood to the tissues that need creatine for absorption. Studies have reported that creatine and creatine analogs could protect tissues from hypoxia and ischemia [35]. Creatinine is important for kidney function itself. Because the CK/PCr system supports ion pumps and metabolite transporters in the kidney, it is responsible for ion balance and the absorption of metabolites from the urine [36]. The results showed that the content of serum creatine in the SPF group was significantly higher than that in the PF group, which may be related to the mild degree of liver damage and the normal creatine synthesis in the SPF group. At the same time, the increase of creatine has a certain protective effect on the oxidative damage of the body tissues, which may be one of the mechanisms of PF processing and detoxification.

### 4.3. Phenylalanine Metabolism

In phenylalanine metabolism, tyrosine (C00082), hippuric acid (C01586), and phenylacetylglycine (C05598) were the downstream metabolites, and their relative content had significant changes. Tyrosine is a metabolite of phenylalanine, which is catalyzed by phenylalanine hydroxylase [37]. In the absence of p-hydroxyphenylpyruvate oxidase and tyrosine converting enzyme, the increase of p-hydroxyphenylpyruvate and tyrosine in the body leads to a sharp increase in the above two amino acids in the blood, resulting in tyrosine poisoning, which is harmful to multiple organs such as liver and kidney [38]. In addition, it was reported that the contents of phenylacetylglycine and hippuric acid are closely related to phospholipid diseases [39, 40]. Phospholipid disease is a lipid storage disorder. Excessive phospholipids are stored in liver cells, lymphocytes, and other cells, resulting in a variety of tissue damage [41, 42]. Due to the content of metabolite tyrosine decreased, hippuric acid and phenylacetylglycine may increase compensably. Studies have reported that there may be some one sidedness in characterizing phospholipid storage disorders from the concentration of serum hippuric acid and phenylacetylglycine. The ratio of phenylacetylglycine/hippuric acid can be used as a biomarker of phospholipid disease [43]. This study found that the ratio of phenylacetylglycine/hippuric acid in the SPF group was lower than that in the PF group, which was closer to that in normal rats. Therefore, the mechanism of SPF toxicity reduction may be related to the decrease of tyrosine content and phenylacetylglycine/hippuric acid ratio in the phenylalanine metabolism pathway.

### 4.4. Linoleic Acid Metabolism

Linoleic acid metabolism was closely related to physiological states such as inflammatory response and hormone production [44]. Linoleic acid (18:2*ω*6) is the precursor of essential fatty acids and long-chain polyunsaturated fatty acids. As the parent compound of the *ω*6PUFA family, linoleic acid can be extended and desaturated to other biologically activated *ω*6PUFA, such as *γ*-linolenic acid (18:3*ω*6) and arachidonic acid (20:4*ω*6) [45]. Arachidonic acid is present in all mammalian cells and is one of the most abundant polyunsaturated fatty acids in human tissues. It is very important in the normal metabolic functions of cells and tissues [46]. However, when excessive eicosanoid acid is continuously produced, arachidonic acid can be converted into a variety of biologically active compounds called eicosanoids, such as prostaglandins and leukotrienes [47, 48]. These eicosanoids are effective mediators of inflammation and are related to the pathogenesis of many diseases [49]. This study found that, compared with the metabolites of rats in the PF group, the contents of linoleic acid and arachidonic acid in the metabolites of rats were significantly reduced in the SPF group. Therefore, the attenuation mechanism of SPF may be related to downregulating the metabolism of linoleic acid and arachidonic acid, reducing liver and kidney damage caused by inflammation.

## 5. Conclusion

In this study, metabolomics was used to study the difference of endogenous metabolites between PF and SPF in rats. Studies have shown that the mechanism of the detoxification of PF by salt processing may be related to the regulation of glycerophospholipid metabolism, phenylalanine, tyrosine, and tryptophan biosynthesis, arginine and proline metabolism, phenylalanine metabolism, and linoleic acid metabolism. By inhibiting the production of metabolites (1-acyl-sn-glycero-3-phosphocholine, sn-glycero-3-phosphocholine, tyrosine, arginine, linoleic acid, arachidonic acid, and phenylacetylglycine/hippuric acid ratio) and upregulating the expression of creatine, we could reduce the inflammatory response induced by PF, so as to achieve the purpose of detoxification after processing.

## Figures and Tables

**Table 1:** Response stability of internal standard in QC sample.

Internal standard	Positive			Negative		
	*m*/*z*	RT	RSD_QC_ (%)	*m*/*z*	RT	RSD_QC_ (%)
L-2-Chlorophenylalanine	200.05	176.49	1.60	198.03	167.97	11.13

**Table 2:** Differential metabolites in positive mode.

No.	Metabolites	KEGG	RT	*m*/*z*	VIP	*P* value	Fold change
1	Creatinine	C00791	34.12	114.07	2.18	0.00107	1.60436
2	Creatine	C00300	34.92	132.08	1.62	0.04020	1.46332
3	Trigonelline	C01004	34.67	138.05	1.71	0.01403	1.45140
4	Dibutyl phthalate	C14214	451.20	279.16	1.86	0.01023	1.10582
5	Tyrosine	C00082	101.32	182.08	1.80	0.00927	0.70458
6	Palmitoylethanolamide	C16512	480.41	300.29	1.61	0.02323	1.44999
7	L-Palmitoylcarnitine	C02990	408.62	400.34	2.11	0.00050	0.47849
8	1-Acyl-sn-glycero-3-phosphocholine	C04230	401.93	568.34	2.25	0.00018	0.74952
9	Aminocaproic acid	C02378	129.37	132.10	1.18	0.02810	0.60651
10	Xanthine	C00385	71.28	153.04	1.49	0.01499	0.44558
11	sn-Glycero-3-phosphocholine	C00670	31.44	258.11	1.89	0.03563	0.57238
12	Arginine	C00062	29.84	197.10	1.62	0.03912	0.78833

**Table 3:** Metabolites in negative mode.

No.	Metabolites	KEGG	RT	*m*/*z*	VIP	*P* value	Fold change
1	Tryptophan	C00078	164.60	203.08	1.89	0.00072	0.58290
2	Phenylacetylglycine	C05598	166.25	192.07	1.60	0.03150	2.21871
3	Norleucine	C01933	60.75	130.09	1.36	0.04702	0.68770
4	*β*-Guanidinopropionic acid	C03065	34.75	130.06	1.60	0.01497	1.56198
5	Linoleic acid	C01595	477.29	279.23	1.44	0.02498	0.67525
6	Xanthine	C00385	69.70	151.03	1.29	0.01471	0.45979
7	Arachidonic acid	C00219	470.30	303.23	1.75	0.00205	0.58638
8	Tyrosine	C00082	60.02	180.07	1.41	0.02640	0.72039
9	Oleic acid	C00712	513.97	281.25	1.42	0.02952	0.75543
10	Hippuric acid	C01586	152.26	178.05	2.00	0.00561	4.68205
11	Creatinine	C00791	55.56	112.05	1.98	0.00012	1.55002
12	3,7-Dimethyluric acid	C16360	29.18	195.05	1.63	0.01541	1.76108

**Table 4:** Metabolic pathway analysis in positive mode.

Pathway	Number	Raw *p*	Impact	Compound and KEGG ID
Glycerophospholipid metabolism	2	0.031437	0.0655	1-Acyl-sn-glycero-3-phosphocholine cpd:C04230; sn-glycero-3-phosphocholine cpd:C00670
Phenylalanine, tyrosine, and tryptophan biosynthesis	1	0.031463	0.5	Tyrosine cpd:C00082
Arginine and proline metabolism	2	0.03477	0.06998	Arginine cpd:C00062; creatine cpd:C00300
Aminoacyl-tRNA biosynthesis	2	0.05338	0	Arginine cpd:C00062; tyrosine cpd:C00082
Ubiquinone and other terpenoid-quinone biosyntheses	1	0.069514	0	Tyrosine cpd:C00082
Phenylalanine metabolism	1	0.091683	0	Tyrosine cpd:C00082
Arginine biosynthesis	1	0.10619	0.07614	Arginine cpd:C00062
Ether lipid metabolism	1	0.14845	0	Glycerophosphocholine cpd:C00670
Glycine, serine, and threonine metabolism	1	0.24004	0	Creatine cpd:C00300
Fatty acid degradation	1	0.27049	0	L-Palmitoylcarnitine cpd:C02990
Tyrosine metabolism	1	0.28822	0.13972	Tyrosine cpd:C00082
Purine metabolism	1	0.41649	0.02966	Xanthine cpd:C00385

**Table 5:** Metabolic pathway analysis in negative mode.

Pathway	Number	Raw *p*	Impact	Compound and KEGG ID
Phenylalanine metabolism	3	8.13E-05	0	Tyrosine cpd:C00082; hippuric acid cpd:C01586; phenylacetylglycine cpd:C05598
Biosynthesis of unsaturated fatty acids	3	0.0023684	0	Oleic acid cpd:C00712; linoleic acid cpd:C01595; arachidonic acid cpd:C00219
Phenylalanine, tyrosine, and tryptophan biosynthesis	1	0.031463	0.5	Tyrosine cpd:C00082
Linoleic acid metabolism	2	0.039185	1	Linoleic acid cpd:C01595; arachidonic acid cpd:C00219
Aminoacyl-tRNA biosynthesis	2	0.05338	0	Tryptophan cpd:C00078; tyrosine cpd:C00082
Ubiquinone and other terpenoid-quinone biosyntheses	1	0.069514	0	Tyrosine cpd:C00082
Caffeine metabolism	1	0.091683	0	3,7-Dimethyluric acid c pd:C16360
Arachidonic acid metabolism	1	0.25235	0.33292	Arachidonic acid cpd:C00219
Tryptophan metabolism	1	0.28236	0.14305	Tryptophan cpd:C00078
Tyrosine metabolism	1	0.28822	0.13972	Tyrosine cpd:C00082
Purine metabolism	1	0.41649	0.02966	Xanthine cpd:C00385

## Data Availability

The data used to support the findings of this study are available from the corresponding author upon request.

## Abbreviations

ANOVA: Analysis of variance
COX-2: Cyclooxygenase type 2
CTR: Centralization
CV: Coefficient of variation
HE: Hematoxylin-eosin
IDA: Information-dependent acquisition
KEGG: Kyoto Encyclopedia of Genes and Genomes
LPC: Lysophosphatidylcholine
OPLS-DA: Orthogonal Partial Least Squares Method-Discriminant Analysis
PCA: Principal component analysis
PF: Psoraleae Fructus
QC: Quality control
RSD: Relative standard deviation
SD: Standard deviation
SPF: Salt-processing Psoraleae Fructus
TIC: Total ion current
UVA: Univariate analysis
VIP: Variable importance in the projection.

## Supplementary Materials

Supplementary Materials Supplementary Table 1: possible differential metabolites identified by secondary mass spectrometry in positive mode. Supplementary Table 2: possible differential metabolites identified by secondary mass spectrometry in negative mode. Supplementary Table 3: possible differential metabolites identified by the HDMB and KEGG databases in positive mode. Supplementary Table 4: possible differential metabolites identified by the HDMB and KEGG databases in negative mode. Supplementary Figure 1: BPC diagrams of all QC samples. (A) In positive mode. (B) In negative mode. The EIC diagram of the internal standard L-2-chlorophenylalanine in the QC sample. (C) In positive mode. (D) In negative mode.
