# Supplementary material for: UPLC-MS/MS-Based Rat Serum Metabolomics Reveals the Detoxification Mechanism of Psoraleae Fructus during Salt Processing
Source: Evid Based Complement Alternat Med. 2021 Sep 14;2021:5597233. doi: 10.1155/2021/5597233 (PMC8457953; doi:10.1155/2021/5597233)
Supplement: Supplementary Materials — Supplementary Table 1: possible differential metabolites identified by secondary mass spectrometry in positive mode. Supplementary Table 2: possible differential metabolites identified by secondary mass spectrometry in negative mode. Supplementary Table 3: possible differential metabolites identified by the HDMB and KEGG databases in positive mode. Supplementary Table 4: possible differential metabolites identified by the HDMB and KEGG databases in negative mode. Supplementary Figure 1: BPC diagrams of all QC samples. (A) In positive mode. (B) In negative mode. The EIC diagram of the internal standard L-2-chlorophenylalanine in the QC sample. (C) In positive mode. (D) In negative mode. [file 5597233.f1.docx]

**Supplementary Table 1. Possible differential metabolites identified by secondary mass spectrometry in positive mode**

| No. | R.T. | Compounds | m/z | VIP | P-VALUE | FOLD CHANGE |
| --- | --- | --- | --- | --- | --- | --- |
| 1 | 34.11520 | CREATININE | 114.066 | 2.183122 | 0.001066 | 1.604358 |
| 2 | 34.91940 | CREATINE | 132.0766 | 1.624213 | 0.040196 | 1.463315 |
| 3 | 421.17750 | CETRIMONIUM | 284.3305 | 1.549719 | 0.041294 | 1.085793 |
| 4 | 451.44750 | Phthalic anhydride | 149.0232 | 1.902316 | 0.007946 | 1.09593 |
| 5 | 34.66505 | TRIGONELLINE | 138.0548 | 1.707063 | 0.014029 | 1.451399 |
| 6 | 451.20100 | Dibutyl phthalate | 279.1585 | 1.860553 | 0.01023 | 1.105824 |
| 7 | 400.31700 | LysoPE(22:6(4Z,7Z,10Z,13Z,16Z,19Z)/0:0) | 526.2914 | 2.065255 | 0.001531 | 0.742003 |
| 8 | 378.96750 | Myristoylcarnitine | 372.3098 | 2.017469 | 0.00275 | 0.603479 |
| 9 | 101.32450 | Tyrosine | 182.0809 | 1.796007 | 0.009268 | 0.704582 |
| 10 | 435.13300 | DL-Stearoylcarnitine | 428.3723 | 1.7961 | 0.003103 | 0.56071 |
| 11 | 176.22550 | Idazoxan | 205.0968 | 2.149131 | 0.001362 | 0.573922 |
| 12 | 480.40750 | Palmitoyl-EA | 300.2889 | 1.606397 | 0.023231 | 1.449995 |
| 13 | 388.98450 | trans-Hexadec-2-enoyl carnitine | 398.3254 | 1.762277 | 0.012963 | 0.579417 |
| 14 | 101.26400 | 2-Aminoacetophenone | 136.0756 | 1.830238 | 0.007294 | 0.698525 |
| 15 | 362.36200 | N-Oleoyl-L-Serine | 370.2941 | 2.016828 | 0.000817 | 0.592237 |
| 16 | 54.17510 | 2-Amino-2-methylbutanoate | 118.086 | 1.544986 | 0.049275 | 0.764701 |
| 17 | 176.22050 | 4-Hydroxyquinoline | 146.0599 | 2.090278 | 0.002339 | 0.567387 |
| 18 | 101.27000 | 9-hydroxy-7E-Nonene-3,5-diynoic acid | 165.0544 | 1.781113 | 0.009932 | 0.699012 |
| 19 | 226.51400 | 3,4-Dehydro-6-hydroxymellein | 193.0493 | 1.698175 | 0.021349 | 1.090162 |
| 20 | 456.01400 | Trimethylaminoacetone | 116.1068 | 1.967631 | 0.003642 | 1.180495 |
| 21 | 433.80200 | PC(22:4(7Z,10Z,13Z,16Z)/0:0) | 572.3695 | 1.854256 | 0.009322 | 0.768114 |
| 22 | 418.78700 | PC(22:6(4E,7E,10E,13E,16E,19E)/0:0)[U] | 568.3356 | 1.621577 | 0.011728 | 0.638005 |
| 23 | 397.65000 | Linoelaidyl carnitine | 424.3409 | 2.14402 | 2.72E-05 | 0.401765 |
| 24 | 408.61900 | Palmitoyl-L-carnitine | 400.341 | 2.105917 | 0.000502 | 0.478492 |
| 25 | 415.29000 | Vaccenyl carnitine | 426.3566 | 2.033594 | 0.001385 | 0.408684 |
| 26 | 420.78000 | Geranyl acetoacetate | 239.1637 | 2.125461 | 0.00063 | 0.605367 |
| 27 | 176.23150 | 1-Benzylimidazole | 159.0914 | 2.047056 | 0.0029 | 0.585415 |
| 28 | 437.56250 | LysoPE(20:2(11Z,14Z)/0:0) | 506.3228 | 2.07439 | 0.020096 | 0.678559 |
| 29 | 408.88200 | PC(17:1(9Z)/0:0) | 508.3384 | 1.687304 | 0.025908 | 0.76208 |
| 30 | 129.37200 | E-aminocaproic acid | 132.1017 | 1.177692 | 0.028104 | 0.606511 |
| 31 | 429.78000 | PC(O-16:1(11Z)/0:0) | 480.3438 | 1.594208 | 0.033757 | 0.79203 |
| 32 | 396.32300 | PC(7:0/O-8:0) | 482.3229 | 1.8809 | 0.007389 | 0.778972 |
| 33 | 495.32900 | (4Z,7Z,10Z,13Z,16Z,19Z)-4,7,10,13,1 6,19-Docosahexaenoic acid | 329.2465 | 2.151157 | 0.01319 | 0.442602 |
| 34 | 190.40700 | Octylamine | 130.1589 | 1.692012 | 0.025143 | 1.089525 |
| 35 | 412.09600 | LysoPC(22:5(4Z,7Z,10Z,13Z,16Z)) | 570.3539 | 1.75077 | 0.01283 | 0.7183 |
| 36 | 176.37300 | 4-Chloro-3,5-dimethoxybenzyl alcohol | 203.0473 | 1.635002 | 0.026375 | 1.082492 |
| 37 | 71.27960 | Xanthine | 153.0405 | 1.493815 | 0.014986 | 0.445577 |
| 38 | 185.85700 | Diphenylphosphate | 251.0479 | 1.261864 | 0.036258 | 3.066625 |
| 39 | 401.39900 | LysoPE(0:0/20:4(5Z,8Z,11Z,14Z)) | 502.2916 | 1.766568 | 0.014493 | 0.812439 |
| 40 | 429.78050 | 24,24-difluoro-1alpha,25-dihydroxy-26,27-dimethylvitamin D3 | 481.3468 | 1.695529 | 0.017626 | 0.757487 |
| 41 | 31.44245 | sn-glycero-3-Phosphocholine | 258.1095 | 1.88853 | 0.035631 | 0.572377 |
| 42 | 401.67700 | Met Arg Arg Gln | 590.3193 | 2.074386 | 0.001263 | 0.812233 |
| 43 | 29.83800 | L-Arginine | 197.1006 | 1.623019 | 0.039116 | 0.788327 |

**Supplementary Table 2. Possible differential metabolites identified by secondary mass spectrometry in negative mode**

| No. | R.T. | Compounds | m/z | VIP | P-VALUE | FOLD CHANGE |
| --- | --- | --- | --- | --- | --- | --- |
| 1 | 164.60100 | Tryptophan | 203.08275 | 1.88995 | 0.00072 | 0.58290 |
| 2 | 166.25200 | Phenylacetylglycine | 192.06682 | 1.60080 | 0.03150 | 2.21871 |
| 3 | 60.75235 | NORLEUCINE | 130.08749 | 1.35696 | 0.04702 | 0.68770 |
| 4 | 34.75365 | 3-Guanidinopropanoate | 130.06231 | 1.59886 | 0.01497 | 1.56198 |
| 5 | 416.62700 | 1-Palmitoyl Lysophosphatidic Acid | 409.23615 | 1.67483 | 0.00487 | 0.78238 |
| 6 | 477.29150 | 9Z,12Z-Linoleic acid | 279.23280 | 1.43923 | 0.02498 | 0.67525 |
| 7 | 69.70055 | Xanthine | 151.02638 | 1.28607 | 0.01471 | 0.45979 |
| 8 | 198.03300 | 3-Indoxylsulfate | 212.00241 | 1.78680 | 0.00128 | 2.19858 |
| 9 | 470.30100 | Arachidonic acid | 303.23297 | 1.74651 | 0.00205 | 0.58638 |
| 10 | 456.94000 | Docosahexanoic acid | 327.23302 | 1.85598 | 0.02597 | 0.48171 |
| 11 | 491.90300 | ponasterone A | 463.30678 | 1.32272 | 0.02968 | 1.68427 |
| 12 | 429.99850 | Confertifoline | 233.15465 | 1.31929 | 0.04685 | 1.08056 |
| 13 | 60.01915 | L-Tyrosine | 180.06678 | 1.41042 | 0.02640 | 0.72039 |
| 14 | 497.90000 | JWH 200 2'-naphthyl isomer | 383.17480 | 1.61387 | 0.00796 | 1.16539 |
| 15 | 370.68600 | Myristic Acid Alkyne | 223.17028 | 1.44464 | 0.01979 | 0.60364 |
| 16 | 513.96600 | Oleic acid | 281.24847 | 1.41942 | 0.02952 | 0.75543 |
| 17 | 152.25600 | HIPPURATE | 178.05112 | 1.99768 | 0.00561 | 4.68205 |
| 18 | 119.44150 | Trichloroacetic acid | 160.89713 | 2.16228 | 0.00002 | 272.35118 |
| 19 | 397.93500 | Phosphatidylcholine lyso 18:2 | 578.34693 | 1.22741 | 0.03539 | 0.63895 |
| 20 | 55.55900 | Creatinine | 112.05180 | 1.97595 | 0.00012 | 1.55002 |
| 21 | 29.17790 | 3,7-Dimethyluric acid | 195.05105 | 1.62992 | 0.01541 | 1.76108 |
| 22 | 496.08000 | Dihomo-alpha-linolenic acid (20:3(n-3)) | 305.24858 | 1.26683 | 0.02711 | 0.54701 |
| 23 | 28.94725 | Phosphoric acid | 96.96966 | 1.52645 | 0.01189 | 1.71988 |
| 24 | 400.48900 | Phosphatidylethanolamine lyso 20:4 | 500.27821 | 1.65386 | 0.00518 | 0.83591 |

**Supplementary Table 3. Possible differential metabolites identified by the HDMB and KEGG databases in positive mode**

| No. | Compounds | Match | HMDB | PubChem | KEGG | Comment |
| --- | --- | --- | --- | --- | --- | --- |
| 1 | CREATININE | Creatinine | HMDB0000562 | 588 | C00791 | 1 |
| 2 | CREATINE | Creatine | HMDB0000064 | 586 | C00300 | 1 |
| 3 | CETRIMONIUM |  |  |  |  | 0 |
| 4 | Phthalic anhydride |  |  |  |  | 0 |
| 5 | TRIGONELLINE | Trigonelline | HMDB0000875 | 5570 | C01004 | 1 |
| 6 | Dibutyl phthalate | Dibutyl phthalate | HMDB0033244 | 3026 | C14214 | 1 |
| 7 | LysoPE(22:6(4Z,7Z,10Z,13Z,16Z,19Z)/0:0) | LysoPE(22:6(4Z,7Z,10Z,13Z,16Z,19Z)/0:0) | HMDB0011526 | 52925132 |  | 1 |
| 8 | Myristoylcarnitine | Tetradecanoylcarnitine | HMDB0005066 | 53477791 |  | 1 |
| 9 | Tyrosine | L-Tyrosine | HMDB0000158 | 6057 | C00082 | 1 |
| 10 | DL-Stearoylcarnitine |  |  |  |  | 0 |
| 11 | Idazoxan |  |  |  |  | 0 |
| 12 | Palmitoyl-EA | Palmitoylethanolamide | HMDB0002100 | 4671 | C16512 | 1 |
| 13 | trans-Hexadec-2-enoyl carnitine | trans-Hexadec-2-enoyl carnitine | HMDB0006317 | 53477817 |  | 1 |
| 14 | 2-Aminoacetophenone | 2-Aminoacetophenone | HMDB0032628 | 11952 |  | 1 |
| 15 | N-Oleoyl-L-Serine |  |  |  |  | 0 |
| 16 | 2-Amino-2-methylbutanoate |  |  |  |  | 0 |
| 17 | 4-Hydroxyquinoline |  |  |  |  | 0 |
| 18 | 9-hydroxy-7E-Nonene-3,5-diynoic acid |  |  |  |  | 0 |
| 19 | 3,4-Dehydro-6-hydroxymellein |  |  |  |  | 0 |
| 20 | Trimethylaminoacetone | Trimethylaminoacetone | HMDB0012296 | 151806 |  | 1 |
| 21 | PC(22:4(7Z,10Z,13Z,16Z)/0:0) |  |  |  |  | 0 |
| 22 | PC(22:6(4E,7E,10E,13E,16E,19E)/0:0)[U] |  |  |  |  | 0 |
| 23 | Linoelaidyl carnitine | Linoelaidyl carnitine | HMDB0006461 | 53477834 |  | 1 |
| 24 | Palmitoyl-L-carnitine | L-Palmitoylcarnitine | HMDB0000222 | 11953816 | C02990 | 1 |
| 25 | Vaccenyl carnitine | Vaccenyl carnitine | HMDB0006351 | 53477830 |  | 1 |
| 26 | Geranyl acetoacetate |  |  |  |  | 0 |
| 27 | 1-Benzylimidazole |  |  |  |  | 0 |
| 28 | LysoPE(20:2(11Z,14Z)/0:0) | LysoPE(20:2(11Z,14Z)/0:0) | HMDB0011513 | 52925140 |  | 1 |
| 29 | PC(17:1(9Z)/0:0) |  |  |  |  | 0 |
| 30 | E-aminocaproic acid | Aminocaproic acid | HMDB0001901 | 564 | C02378 | 1 |
| 31 | PC(O-16:1(11Z)/0:0) |  |  |  |  | 0 |
| 32 | PC(7:0/O-8:0) |  |  |  |  | 0 |
| 33 | (4Z,7Z,10Z,13Z,16Z,19Z)-4,7,10,13,1 6,19-Docosahexaenoic acid |  |  |  |  | 0 |
| 34 | Octylamine |  |  |  |  | 0 |
| 35 | LysoPC(22:5(4Z,7Z,10Z,13Z,16Z)) | LysoPC(22:5(4Z,7Z,10Z,13Z,16Z)) | HMDB0010402 | 53480473 | C04230 | 1 |
| 36 | 4-Chloro-3,5-dimethoxybenzyl alcohol |  |  |  |  | 0 |
| 37 | Xanthine | Xanthine | HMDB0000292 | 1188 | C00385 | 1 |
| 38 | Diphenylphosphate |  |  |  |  | 0 |
| 39 | LysoPE(0:0/20:4(5Z,8Z,11Z,14Z)) | LysoPE(0:0/20:4(5Z,8Z,11Z,14Z)) | HMDB0011487 | 53480936 |  | 1 |
| 40 | 24,24-difluoro-1alpha,25-dihydroxy-26,27-dimethylvitamin D3 |  |  |  |  | 0 |
| 41 | sn-glycero-3-Phosphocholine | Glycerophosphocholine | HMDB0000086 | 71920 | C00670 | 1 |
| 42 | Met Arg Arg Gln |  |  |  |  | 0 |
| 43 | L-Arginine | L-Arginine | HMDB0000517 | 6322 | C00062 | 1 |

Note: Only the compounds that could be accurately matched were selected as differential metabolites and were highlighted in blue in the table.

**Supplementary Table 4. Possible differential metabolites identified by the HDMB and KEGG databases in negative mode**

| No. | Compounds | Match | HMDB | PubChem | KEGG | Comment |
| --- | --- | --- | --- | --- | --- | --- |
| 1 | Tryptophan | L-Tryptophan | HMDB0000929 | 6305 | C00078 | 1 |
| 2 | Phenylacetylglycine | Phenylacetylglycine | HMDB0000821 | 68144 | C05598 | 1 |
| 3 | NORLEUCINE | L-Norleucine | HMDB0001645 | 21236 | C01933 | 1 |
| 4 | 3-Guanidinopropanoate | Beta-Guanidinopropionic acid | HMDB0013222 | 67701 | C03065 | 1 |
| 5 | 1-Palmitoyl Lysophosphatidic Acid |  |  |  |  | 0 |
| 6 | 9Z,12Z-Linoleic acid | Linoleic acid | HMDB0000673 | 5280450 | C01595 | 1 |
| 7 | Xanthine | Xanthine | HMDB0000292 | 1188 | C00385 | 1 |
| 8 | 3-Indoxylsulfate | Indoxyl sulfate | HMDB0000682 | 10258 |  | 1 |
| 9 | Arachidonic acid | Arachidonic acid | HMDB0001043 | 444899 | C00219 | 1 |
| 10 | Docosahexanoic acid |  |  |  |  | 0 |
| 11 | ponasterone A |  |  |  |  | 0 |
| 12 | Confertifoline |  |  |  |  | 0 |
| 13 | L-Tyrosine | L-Tyrosine | HMDB0000158 | 6057 | C00082 | 1 |
| 14 | JWH 200 2'-naphthyl isomer |  |  |  |  | 0 |
| 15 | Myristic Acid Alkyne |  |  |  |  | 0 |
| 16 | Oleic acid | Oleic acid | HMDB0000207 | 445639 | C00712 | 1 |
| 17 | HIPPURATE | Hippuric acid | HMDB0000714 | 464 | C01586 | 1 |
| 18 | Trichloroacetic acid | Trichloroacetic acid | HMDB0042048 |  |  | 1 |
| 19 | Phosphatidylcholine lyso 18:2 |  |  |  |  | 0 |
| 20 | Creatinine | Creatinine | HMDB0000562 | 588 | C00791 | 1 |
| 21 | 3,7-Dimethyluric acid | 3,7-Dimethyluric acid | HMDB0001982 | 83126 |  | 1 |
| 22 | Dihomo-alpha-linolenic acid (20:3(n-3)) |  |  |  |  | 0 |
| 23 | Phosphoric acid | Phosphoric acid | HMDB0002142 | 1004 | C00009 | 1 |
| 24 | Phosphatidylethanolamine lyso 20:4 |  |  |  |  | 0 |

Note: Only the compounds that could be accurately matched were selected as differential metabolites and were highlighted in blue in the table.


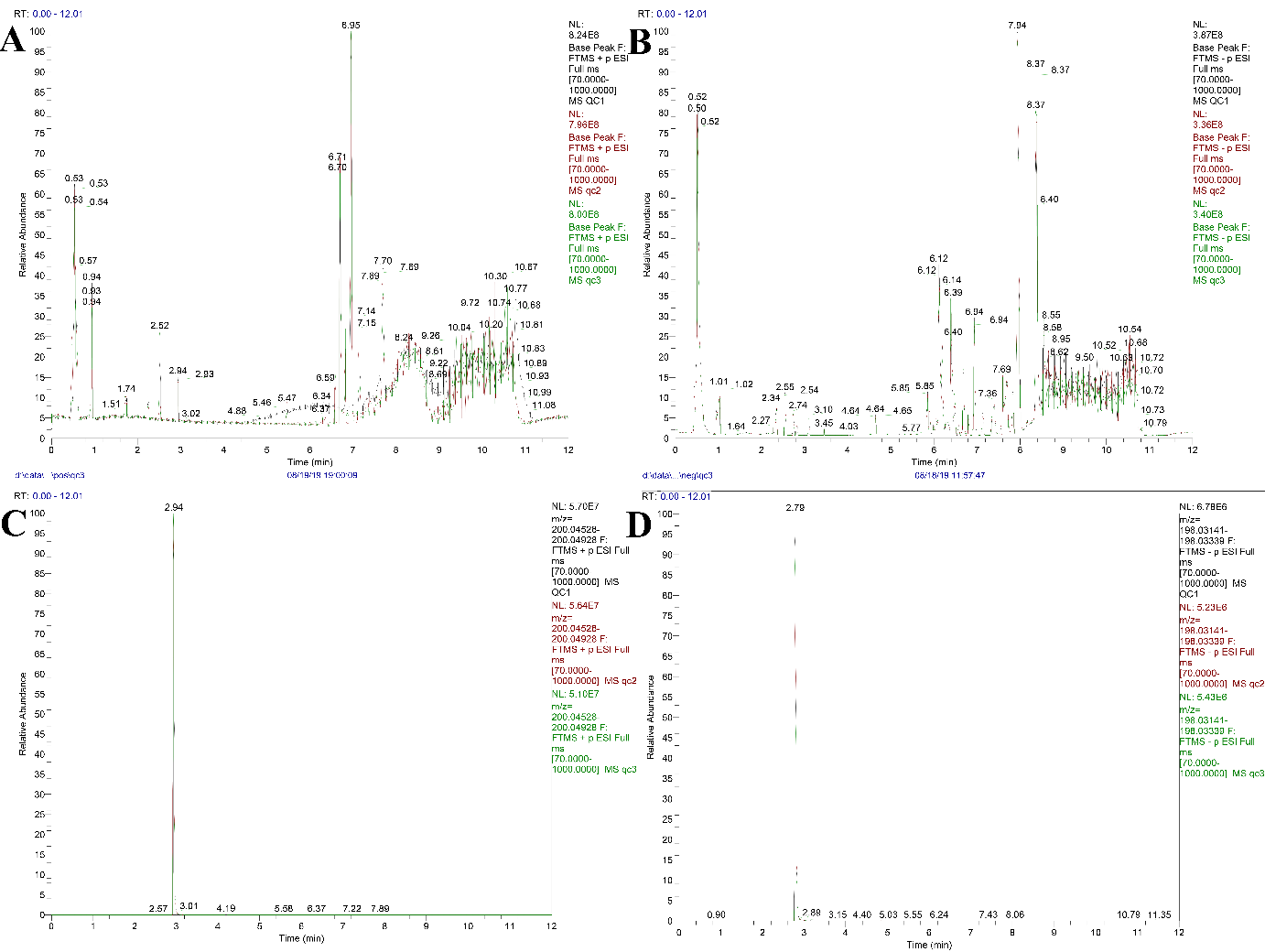


Supplementary Figure 1. BPC diagrams of all QC samples.

(A) In positive mode. (B) In negative mode. The EIC diagram of the internal standard L-2-chlorophenylalanine in the QC sample. (C) In positive mode. (D) In negative mode.
